# Resilience in Stroke Patients: A Concept Analysis

**DOI:** 10.3390/healthcare10112281

**Published:** 2022-11-14

**Authors:** Huey-Yeu Yan, Hung-Ru Lin

**Affiliations:** 1 School of Nursing, National Taipei University of Nursing and Health Sciences, Taipei City 112303, Taiwan; 2 Department of Nursing, University of Kang Ning, Taipei City 114311, Taiwan

**Keywords:** stroke patients, resilience, concept analysis, measurement instrument

## Abstract

(1) **Background:** Resilience enables individuals to develop positive coping strategies when faced with adversity. However, knowledge on resilience in stroke patients remains limited, and resilience assessment methods continue to be debated. (2) **Aim:** To perform a concept analysis of resilience in stroke patients. (3) **Methods:** The Walker and Avant approach to concept analysis was adopted. Searches were performed in the PubMed, Cumulative Index to Nursing and Allied Health Literature (CINAHL), Medical Literature Analysis and Retrieval System Online (MEDLINE), and Chinese Electronic Periodical Services (CEPS) databases and grey literature using the keywords “stroke patient”, “resilience”, and “concept analysis”. (4) **Results:** The defining attributes of resilience in stroke patients were classified into internal personality traits and external environmental support. Antecedents included physical, mental, and familial and social impairments caused by the disease, and the consequences were good adaptation, which included active cooperation with rehabilitation activities, positive thinking, goal setting, and harboring hope for the future. (5) **Conclusion:** Resilience is of a diverse and complex nature. Optimism and positivity, problem-solving ability, and familial and social support may assist in re-adjusting or restoring the balance between physical and mental health when a stroke patient faces adversity. Key factors affecting resilience in stroke patients should be further investigated in future research to assist in the development of effective interventional treatment measures.

## 1. Introduction

Cerebrovascular accidents (CVAs), commonly known as strokes, are a central nervous system disease commonly encountered in clinical practice and the main cause of death and disability worldwide. According to the 2022 Heart Disease and Stroke Statistics Update released by the American Heart Association, there were 7.08 million deaths attributable to cerebrovascular disease globally in 2020, and stroke accounted for approximately one of every 19 deaths in the United States in 2019, making it the fifth leading cause of death in the country [1]. In Taiwan, stroke has consistently ranked fourth among the top 10 leading causes of death and is estimated to cause more than 10,000 deaths annually [2]. Neurofunctional damage caused by stroke necessitates long-term treatment and rehabilitation in the majority of stroke patients; from an economic point of view, this not only reduces the quality of life (QOL), but also adds a heavy burden to families, communities and society [3,4,5]. Therefore, greater attention should be paid to the threat and damage caused by stroke to the life and health of humans.

Resilience refers to the ability to “bounce back” and thrive in the face of adversity. It enables the improvement in the mental health and QOL of individuals [6,7] and is also an adaptive ability that attenuates the impact of adverse events when individuals face the threat of disease or are required to undergo long-term treatment [8,9]. In recent years, there has been a steady increase in multidisciplinary studies and applications of resilience. The importance of resilience has also been increasingly emphasized across different academic fields. However, the majority of research in the nursing discipline has focused on resilience in adolescents and the elderly. There is a clear lack of knowledge of resilience in stroke patients, and differences also exist in the understanding of resilience among most people [7,10,11,12,13]. In clinical practice, it has been observed that certain patients are able to adapt well when faced with disease, despite encountering severe difficulties and obstacles. However, the role played by resilience in the life and environment of stroke patients and the protective factors that counteract risk factors and promote the resolution of difficult situations or perseverance and optimism remain unclear.

According to the Merriam-Webster dictionary [14], a stroke is defined as the sudden impairment or loss of consciousness, sensation, and voluntary motion that is caused by rupture or obstruction (such as by a clot) of a vessel supplying blood to the brain, and is accompanied by permanent damage of brain tissue. A patient is defined as an individual awaiting or under medical care and treatment. There are two meanings of resilience: 1. The capability of a strained body to recover its size and shape after deformation caused especially by compressive stress; 2. An ability to recover from or adjust easily to misfortune or change. Based on the aforementioned dictionary definitions, the resilience of a stroke patient can be defined as the ability of an individual to recover from or adjust to stress, misfortune, or change while receiving medical care and treatment for damage to physical function caused by brain damage.

The term “resilience” originated from the disciplines of psychiatry and psychological counselling and has been studied and described since the 1950s. In the past, the phenomenon in which a group of children who grew up in a high-risk environment were able to overcome their adversity and become healthy, competent adults was mostly described as invulnerability, coping, or stress resistance [15,16,17,18]. During the 1970s, resilience gradually came to be considered an outcome of positive adaptation, with its content including possible risk and protective factors that favor individual adjustment. When the number of protective factors is increased, resilience against external challenges becomes stronger [19,20]. In the 1980s, resilience was regarded as an intensifier that assisted in the process of adjustment when an individual experienced adversity [21,22,23]. With the surge of positivist philosophy in the 1990s, the definition of resilience evolved into “a strength that can be evoked and nurtured following the interactions between an individual and the ecosystem (including family and community)” [24,25,26,27]. As per the development process described above, the definition of resilience is now generally accepted as “a process or outcome in which the strength or protective factors generated after the interactions of an individual with the environment promote the shift toward positive adaptation in the face of adversity” [28,29,30].

With knowledge transposition, differences have emerged in the understanding of resilience among various professional fields. The American Psychological Association defines resilience as “the process and outcome of successfully adapting to difficult or challenging life experiences, especially through mental, emotional, and behavioral flexibility and adjustment to external and internal demands [31].” On the website of the United States Department of Education, resilience is described as “the process of adapting well in the face of adversity, trauma, tragedy, threats or even significant sources of stress, and also an ability that helps students to cope with negative psychological influences [32].” Nursing researchers have mostly described resilience as “certain traits or abilities that enable an individual to develop healthy coping strategies when faced with a crisis or stressful situation [33,34,35]”. On the basis of the aforementioned definitions, resilience in stroke patients can be conceptually defined as “the process or outcome of utilizing the strength generated from interactions between an individual and the environment during medical treatment and care for the damage of physical function caused by rupture or obstruction of a vessel supplying blood to the brain to develop healthy coping strategies and promote the shift toward positive adaptation.”

Empowerment, self-efficacy, and adaptation are three commonly mentioned concepts that are similar to resilience [36,37]. According to the online Cambridge Dictionary, empowerment is defined as giving someone official authority or the freedom to do something [38]. In other words, it is the process or outcome of an individual receiving certain authority and resources to control their living abilities [39]. However, a key aspect of empowerment is that the enhancement of one’s ability to change their situation is brought about through external assistance [40,41]. The concept of self-efficacy, first proposed by the renowned psychologist Albert Bandura in 1986, refers to an individual’s belief in their capacity to execute behaviors necessary to produce specific performance attainments [42]. It emphasizes the self-judgement of capacity and is unrelated to the skills possessed by the individual [43]. Adaptation refers to the state or process of harmony and balance resulting from an individual’s effective overcoming of or acclimatization to various situations in the environment and subsequent engagement in interactions with the surrounding environment [44], with its key aspect being the process of self-change and outcome of balancing. Considering that resilience is intrinsically complex, diverse, and dynamic; nearly encompasses the three concepts described above; and is linked to various professional fields [11,45,46], it is suitable for use as a reference for the formulation of clinical guidelines for the care of stroke patients.

The present study therefore aimed to utilize the conceptual analysis model of Walker and Avant to examine the resilience in stroke patients and provide required clarity to the concept. Other than clarifying the development history, core composition and situational definition of the concept, we also hope to expand our current understanding and establish key knowledge on the resilience potential of patients, thereby providing a reference for the formulation of clinical health care guidelines [47].

## 2. Materials and Methods

**Figure 1:**
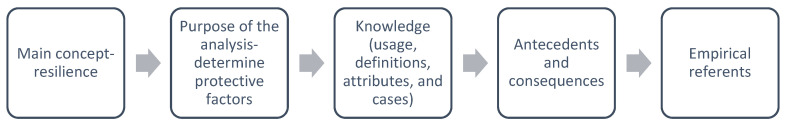
Main steps of the concept analysis of resilience in stroke patients.

Concept analysis is a process of examining the basic elements of a concept; it can be useful in refining ambiguous concepts and constructing research instruments [14]. To investigate the components of the concept of resilience in stroke patients, we adopted the Walker and Avant approach to concept analysis, comprising the following steps:

1. Select a concept: choose a concept of interest, usually the substantial and important topic encountered in clinical works.

2. Determine the purpose of the analysis: focusing on the aims and intention of how to use the collected results. What is the importance of this concept? Why analyze the concept?

3. Identify all uses of the concept: identify as many uses of the concept as necessary, covering all possible fields of knowledge related to it.

4. Determine the defining attributes: identify the cluster of attributes of characteristics that are most frequently associated with the concept; it is crucial to the concept analysis, through it exploiting the essence of the concept.

5. Construct a model case: identify a model case that demonstrates all the defining attributes of the concept. The case was constructed based on real clinical observations.

6. Construct an additional case: identify borderline case (containing most but not all the defining attributes), related care (containing similar but not the same defining attributes) and contrary cases that are not based on said concept. The cases were constructed based on real clinical observations.

7. Identify antecedents and consequences: identify the events or incidents that must occur prior to and after the occurrence of the concept.

8. Define empirical referents: describe empirical references that facilitate recognition or measurement of the defining characteristics or attributes. Figure 1 shows the main steps of the concept analysis performed in this study [47].

### Data Sources

**Figure 2:**
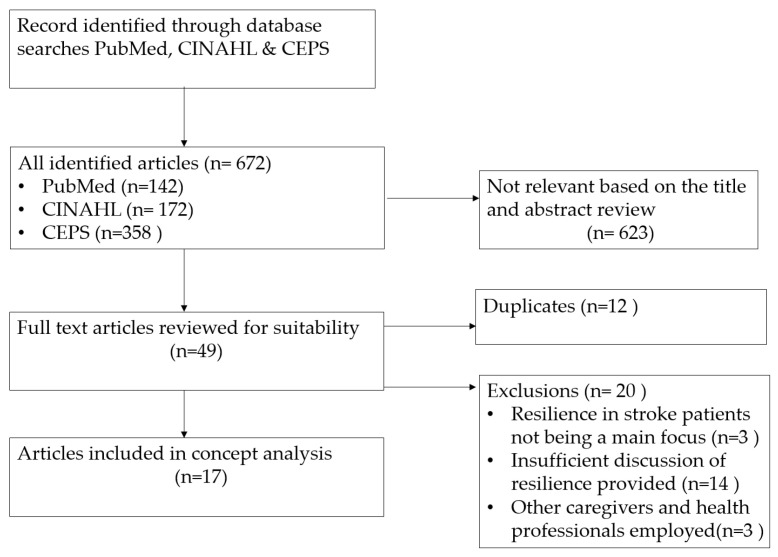
Flow chart of literature search.

Our data sources included Chinese- and English-language journals, books, and the Cambridge Dictionary. Searches were also performed in PubMed, the Cumulative Index to Nursing and Allied Health Literature (CINAHL), the Medical Literature Analysis and Retrieval System Online (MEDLINE), and the Chinese Electronic Periodical Services (CEPS) databases, using the keywords “stroke,” “stroke patient,” “resilience,” and “concept analysis.” The inclusion criteria were as follows: (1) Review articles and empirical studies on resilience in stroke patients; and (2) Articles published between 1950 and 30 May 2022. We further analyzed data from grey literature, which included data published on the websites of governmental agencies and educational institutions. The exclusion criteria were: (1) resilience in stroke patients not being a main focus; (2) insufficient discussion of resilience provided; and (3) other caregivers and health professionals employed (Figure 2).

## 3. Results

Based on the literature-searching strategies described and the inclusion and exclusion criteria, seventeen articles were selected for use in the concept analysis procedure below. In the procedure, numerous attributes or characteristics that are most frequently associated with resilience in stroke patient were identified, and the antecedents and consequences were also identified. Table 1 contains the literature utilized in the concept analysis.

### 3.1. Defining Attributes

A common finding in the related literature is that the main components of resilience are mainly constructed based on major adversity and good adaptation. In individuals who exhibit a high level of resilience, the defining attributes can generally be divided into two main categories. First, the personality traits of such individuals usually include the following: (1) Social competence: including optimism and positivity, flexibility, good communication and interpersonal skills, and the use of humor to cope with stress; (2) Problem-solving skills: actively adopting coping strategies and overcoming difficulties; (3) Autonomy: ability to perceive reality, concern with personal achievement and self-control; and (4) A sense of purpose and future: including control over one’s life (belief in internal control), self-efficacy, expectations and hopes for the future, self-worth, and purpose of life [11,46,48,49,50].

Second, the external environment of the individual, which includes their family, school, community, and workplace, can provide the following: (1) care and support; (2) positive expectations; and (3) opportunities for continuous participation, such as good husband–wife or parent–child relationships, concern from friends, and social support and resources for returning to work [4,10,33,51,52,53]. 

#### 3.1.1. Model Case

Based on the above-mentioned defining attributes or characteristics of resilience, we constructed a model case that included all defining attributes to assist in the understanding of resilience in stroke patients.

Madam Wu, a 50-year-old ovarian cancer patient, suddenly suffered a stroke after undergoing chemotherapy. This led to mild hemiplegia and non-fluent speech, thus resulting in hospitalization for rehabilitation treatment (adversity). Her husband and children visited her in her hospital ward after work or school every day to comfort her and provide encouragement, her mother provided full-time assistance in activities of daily living (familial support), and her best friends frequently visited her and showed concern (social support). Madam Wu, being an optimist, poked fun at herself by joking that experiencing a stroke was luckier than winning the lottery (humor). She believed in the Chinese saying that “a disaster survived is a blessing in store” and that she would completely recover after putting her best effort in rehabilitation (active adoption of coping strategies, harboring positive expectations toward goals). Madam Wu also hoped to return to work soon and continue assisting her husband in his business (looking forward to the future with hope).

#### 3.1.2. Borderline Case

This case possessed most but not all of the defining attributes of resilience in stroke patients.

Mr. Wang, a 40-year-old secondary school teacher, had concomitant hypertension and suffered from a stroke. This led to mild hemiparesis, which resulted in hospitalization for rehabilitation treatment (adversity). His wife, Mrs. Wang, arrived on his ward punctually at 9:00 am daily to accompany him for his rehabilitation session and left thereafter. Mr. Wang felt depressed about suffering a stroke at a young age and viewed himself as useless and a burden to his family. During our interview, he expressed the hope of regaining his health and returning to work soon (harboring hope). He was willing to cooperate with various rehabilitation activities and actively utilized his free time outside of rehabilitation to practice walking in his ward (positive coping strategy).

#### 3.1.3. Related Case

The related case described below did not possess the defining attributes of resilience in stroke patients.

Mr. Lin, a 26-year-old engineer in the electronics industry, was using the computer late one night when he suddenly experienced left-sided hemiplegia caused by a stroke. He was subsequently hospitalized for rehabilitation treatment (adversity). Most of his time was spent alone in his hospital ward and assistance in the activities of daily living was provided by a foreign caregiver. Mr. Lin exhibited feelings of helplessness about being afflicted by a stroke at a young age and was unwilling to talk to others. He expressed resignation regarding his current condition and adopted a passive attitude toward rehabilitation, despite cooperating with the execution of various treatment activities.

#### 3.1.4. Contrary Case

This case was completely contrary to the defining attributes and lacked all core attributes of resilience in stroke patients.

Mr. Chen, a 66-year-old widower who lived alone, suffered from concomitant hypertension and stroke (adversity). During hospitalization, his family members did not visit him, and assistance in activities of daily living was provided by a caregiver. Mr. Chen displayed a poor attitude toward nurses (poor interpersonal relationships and interactions) and frequently grumbled about his children’s unwillingness to visit him (lack of familial support). He felt that there was no point in living as he had to depend on others for everyday tasks (low self-esteem). He often complained of poor physical condition and was unwilling to attend his thrice-weekly physical therapy sessions (poor autonomy). Sometimes, he mumbled to himself that there was no point in exercising as it was completely ineffective. He would spend most of his time lying on his hospital bed and lamenting his lack of luck (lack of problem-solving skills).

### 3.2. Antecedents

Antecedents refer to the causes of the development of resilience, namely, adversity events such as trauma, stress, and disease. Clinical studies have shown that the major challenges and common physical impairments encountered by post-stroke patients include the following: (1) Damage to limb function such as disability and paralysis, which leads to limited mobility and self-care deficit; (2) Speech disorders such as dysphagia, non-fluent speech, or aphasia, which reduce the ability for interpersonal communication and interactions; and (3) Cognitive impairments such as attention deficit, irritability, and memory impairment, which occur in approximately one-third of patients [4,11,12].

Another major category of antecedents was mental impairments, which were often accompanied by negative emotions such as disappointment, misery, nervousness, anxiety, sadness, depression, fear, and anger, along with the manifestation of a negative self-concept and loss of self-esteem [54,55]. The last category was familial and additionally took in social impairments, which included interpersonal difficulties, medical insurance problems, and post-acute care issues. These were caused by changes in personal, familial, and social roles of stroke patients following the interruption of their work life by stroke, which led them to worry about their source of income, future job opportunities, and lack of health care resources [12,13,56,57].

### 3.3. Consequences

Consequences refer to the outcomes of the development of resilience, namely, good adaptation. Previous studies have reported that healthy strategies developed through positive coping with disease by stroke patients promoted the shift toward good adaptation, which includes the following: (1) Personal control: active cooperation with rehabilitation activities, increasing physical activity and enhancing self-care ability, regaining autonomy over the body; (2) Psychological adjustment: changing or adjusting views regarding the disease, exhibiting the ability to be independent and autonomous, and making efforts to reduce the burden of family members with the help of familial and social support, including resources from important people and the health care environment; (3) Personal growth: achieving growth in the face of adversity, shifting toward positive thinking, adopting an optimistic attitude, and facing challenges; and (4) Harboring hope for the future: setting goals and creating meaning and value in life [4,11,12,49,58].

### 3.4. Empirical Referents

**Figure 3:**
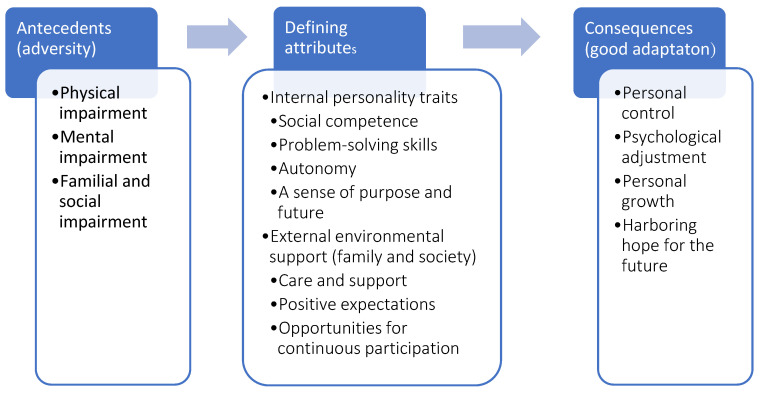
Conceptual model of resilience in stroke patients.

Empirical referents refer to the defining attributes or characteristics that confirm the occurrence of resilience. The antecedents and consequences of the occurrence of resilience in stroke patients and the defining attributes of the demonstration of good resilience can be organized into the following conceptual model (Figure 3). 

Considering that resilience is a dynamic and complex process as well as a self-adjustment ability that changes with time, the assessment methods used differ according to the differences in subjects and purposes or constructivist perspectives. Therefore, there is currently no gold standard instrument for measuring resilience [59,60,61,62]. In our literature search, we observed that the measurement instruments mainly used in clinical practice include the resilience scale (RS), Connor–Davidson Resilience Scale (CD-RISC), brief resilience scale (BRS), and resilience scale for adults (RSA) [7,9,63].

The RS was developed based on adaptability in older adults [64]. Its content was constructed based on data obtained from a qualitative interview study published by the researchers in 1990, and the 25-item scale comprises five factors. Results of the principal component analysis (PCA) of the RS yielded a structure of two factors, namely, personal competence and acceptance of self and life. The 25-item CD-RISC, which defined resilience from another perspective and viewed it as the persistent maintenance of a strong or stable mental health state in the face of adversity, was developed for the purpose of assessing the stress-coping ability of individuals. It also consists of five dimensions, including personal competence, positive acceptance of change, and control [65]. The six-item BRS was developed for assessing resilience traits—the ability to recover from stress or trauma [66]. It is a single-factor instrument with all six items assessing the ability to “bounce back.” Both the RS and CD-RISC merely focus on the assessment of personality traits (e.g., personal ability and control) and exclude important protective factors of interpersonal interactions (e.g., family cohesion, social support, and resources) [67,68], whereas the BRS emphasizes the outcome of resilience assessment while neglecting the essential influencing factors and interaction process [6,8], thus limiting the clinical applicability of these instruments.

The RSA is a self-report scale developed by Hjemdal et al. [69], based on data from previous literature to assess the protective resources that can promote adaptation when adults face psychosocial adversities and health hazards. It is a 33-item scale consisting of six factors, among which four are internal personality traits (perception of self, planned future, social competence, and structured style), while two are external support systems (family cohesion and social resources). Each item consists of both positive and negative attributes to prevent response biases and is scored on a seven-point semantic differential scale ranging from one (strongly disagree) to seven (strongly agree). The total score is obtained by summing the scores of all items, with higher scores indicating higher levels of protective resilience. The questionnaire has demonstrated good psychometric properties, internal consistency (α: 0.74–0.83), retest reliability (r ≥ 0.8), and high levels of convergent and discriminant validity [29,61,70,71]. It has since been translated into multiple languages and used in different populations, including the general population, university students, individuals subjected to trauma, heart disease patients undergoing rehabilitation, and patients with chronic pain [72,73,74,75,76]. Therefore, it may serve as an important assessment tool in clinical practice for the prevention of maladaptation and mental disorders in stroke patients.

## 4. Conclusions

Resilience is an inherently diverse and complex construct. This study utilized the model of Walker and Avant’s concept analysis to examine the resilience in stroke patients. From the literature, the main attributes, such as optimism, self-esteem, interpersonal skills, problem-solving skills, humor, support from family and friends, and returning to the workplace are most frequently associated with the concept. It was revealed that personality traits and the external environment are key influencing factors that enable individuals to demonstrate a good adaptation process or outcome when faced with a major adversity. The results of our analysis revealed that the meaning and value of life created by individuals when faced with adversity determine whether stroke patients who have suffered physical, mental, and familial and social impairments caused by the disease can utilize the strength produced from the interactions of the aforementioned influencing factors during the treatment process to successfully develop healthy coping strategies. Therefore, the enhancement of resilience in stroke patients is a key task for clinical nursing personnel.

Different viewpoints regarding resilience during the development of empirical measurement instruments, particularly in the aspects of positive adjustment or good adaptation, have led to considerable debate among researchers. This has led to a lack of a standard measurement tool for resilience [59,60]. The validity and reliability of the RSA has been well demonstrated in social science and natural science studies, making it applicable to the assessment of protective factors that enable the recovery and maintenance of mental health in individuals who encounter disease, trauma, or stress. Therefore, it may serve as an important assessment tool for clinical nurses to determine the resilience potential of stroke patients in an active manner. Finally, the concept analysis of resilience performed in this study not only provides clarification of the meaning of words or terms to avoid misconception or the overlooking of its true meaning, but can also serve as a reference for the formulation of clinical guidelines for the care of stroke patients.

The study provided a comprehensive analysis of resilience in stroke patients; however, there are some potential limitations. Hundreds of Chinese and English items of literature from the PubMed, CINAHL and CEPS databases were screened to obtain a final list of closely related major references, with minor information extracted from other data sources. Although multicultural databases provided the benefit of multiple dimensional understanding of resilience in stroke patients of various cultures, it could limit certain international academic communication, which is mainly conducted in the English language. As such, it is recommended that a cross-cultural communication strategy be taken into consideration to avoid bias induced by language differences. On the other hand, recent study has revealed that cerebral atrophy is a newly emerging feature of cerebral small vessel diseases, and this atrophy of the gray matter is usually progressive and documented mainly in patients with acute stroke of the lacunar type [77]. The relationship between resilience and cerebral atrophy should be further investigated in future research. One development could be the precise assessment of resilience for lacunar versus non-lacunar acute stroke, since the pathophysiology, prognosis and clinical features of acute small-vessel ischemic strokes are different from other types of cerebral infarcts; and lacunar infarcts are the stroke subtype with the best functional prognosis [78].

## Figures and Tables

**Table 1:** Literature utilized in concept analysis.

Authors	Country	Design	Antecedents	Defining Attributes	Consequences	Empirical References
Tsai et al. (2016) [4]	Taiwan	Qualitative	Impact: loss of physical autonomy, diminishing perception of self-valve, social isolation	Self-encouragement and rethinking, receiving support from family, hope for the future	Adaptation, regaining autonomy after rehabilitation, release from suffering, becoming more positive	Interviews
Wang et al. (2018) [8]	Taiwan	Quantitative	Adversity	Autonomy, interest in things, independence, energy, goal orientation, social support function and family members and friends	Good resilience, self-acceptance	Resilience Scale
Michael (2014) [11]	USA	Literature review	Adverse, physical or psychological challenges	Self-esteem, dispositional attributes (humor, optimistic, positive interpersonal relationships, flexible nature), social support, spirituality, and emotions or outlook, feelings of acceptance and belonging	Good adaptive skills	N/A
Sarre et al. (2014) [12]	UK	Systematic review	Adverse, physical and cognitive impairments, profound effect on survivors’ sense of self and on their relationships	Personal characteristics (determination perseverance, a positive outlook, hope, inner strength, humor) Relationships and structural factors (personal, inter-personal and structural resources, family and friends provide help, belonging, faith)	Practical adjustment, psychosocial recovery, looking forward could provide hope of more favorable possibilities, adjust relationships and structural factors (such as access to health services, employment possibilities and welfare systems)	N/A
Li (2020) [13]	China	Literature review	Adversity, trauma, stress	Emotion, cognition, personality, coping style, self-efficacy, optimism, independence, gratitude, tolerance, humor, sense of responsibility, career, belief, purpose, desire to live, self-confidence, sense of security, family care and care, emotional support, harmonious family interpersonal relationships, friend support	Well-adjusted in the face of negative experiences	N/A
Tseng (2006) [34]	Taiwan	Meta-analysis	Adversity: biological, psychological and social crisis	Positive emotions, intelligence, interpersonal skills, internal a control beliefs, Actual control expectations, self-efficacy, coping with problems, family support, external connection, social support network,	Positive adaptation: maintain good ability include cognitive, psychological, and social well-being	N/A
Ch’Ng et al. (2008) [48]	Australia	Qualitative	Physical and psychological challenge	Active, social and cognitive coping strategies (adopt a problem solving stance, acceptance, moving beyond, information seeking, support from family and friends	Adjustment, acceptance of life changes, engagement in new roles and activities and the presence of social support	Interviews
Erikson et al. (2010) [49]	Sweden	Qualitative	Adversity, psychological distress, social isolation	Inspiration and belonging through acting with others, reality adjustment through acting with others, family supports, workplace for social acting and support,	A process of belonging for integration, get to know oneself, self-compassion	Interviews
Joyce et al. (2018) [50]	Australia	Systematic review and Meta-analysis	Significant challenge or threat to the individual	Mindfulness and/or cognitive and behavioral skills	Quality of adaptation, bounce back	N/A
Shipley et al. (2018) [51]	Australia	Qualitative	Trauma, emotional, losing pre-stroke life construct and relationships	Living with and adapting to stroke sequelae, moving forward, returning to work or study	Gaining a new appreciation for the important things in life, post-traumatic growth	Interviews
Price et al. (2012) [52]	USA	Qualitative	Disability	Positive social support, accessing spirituality, Internal locus of control, building on past successes and a commitment to succeed, and having an action-oriented approach and positive personal goals for the future	Adaptation, getting function back, Overcome the challenges of disability,	Interviews
Zhou et al. (2020) [53]	China	Quantitative	Post-stroke depression, physical and psychosocial factors dysfunction, disability	Intimate friends, good social support	Positive emotions	Connor-Davidson Resilience Scale
Fox et al. (2003) [54]	Australia	Qualitative	Adversity	Humor, perseverance, sense of pride, social support, disposition, competency and self-esteem	Successful adaptation	Interviews
Hunting Pompon et al. (2022) [55]	USA	Literature review	Adversity	Positive self-concept, attitude/outlook, humor, confidence, self-worth, self-efficacy, engaging in new activities and opportunities for social connection, support groups	Positive adaptation, adjustment, and coping abilities	N/A
de Guzman et al. (2012) [56]	Philippines	Qualitative	Trauma	Self-concept, relationships with others, disposition on innate feelings	Accept and to cope with the chronic effects of stroke	Interviews
Wang et al. (2019) [57]	China	Quantitative	Functional disability, psychological distress	Activity of daily living, social support, hope, self-efficacy	Recovering and adapting	Resilience Scale
Chen et al. (2022) [58]	USA	Qualitative	Stress	Positive thoughts, confidence, and support from family and health professionals	Psychological adjustment, from zero to hero	Interviews

## Data Availability

The datasets used and/or analysed during the current study are available from the corresponding author (HL) on reasonable request.
